# Draft Genome Sequence of *Salegentibacter* sp. Strain BDJ18, a Plankton-Associated Bacterium in the Northeast Atlantic Ocean

**DOI:** 10.1128/MRA.00628-21

**Published:** 2021-09-09

**Authors:** Emily J. McDermith, Alexa R. Sterling, Matthew J. Bertin, Bethany D. Jenkins

**Affiliations:** a Department of Cell and Molecular Biology, University of Rhode Island, Kingston, Rhode Island, USA; b Department of Biomedical and Pharmaceutical Sciences, College of Pharmacy, University of Rhode Island, Kingston, Rhode Island, USA; c Graduate School of Oceanography, University of Rhode Island, Narragansett, Rhode Island, USA; Montana State University

## Abstract

*Salegentibacter* sp. strain BDJ18 was isolated from a plankton-associated seawater sample from the northeast Atlantic Ocean. We report its draft genome assembly, which includes genes potentially important for microbial interactions in the marine environment.

## ANNOUNCEMENT

*Salegentibacter* spp., Gram-negative bacteria within *Flavobacteria* that require oxygen and salt for growth, are known to associate with marine phytoplankton (1, 2) and have been isolated from saline habitats, including hypersaline lakes, ocean sediments, and marine animals (1). We report a *Salegentibacter* sp. isolate from the water column plankton community, indicating its potential role in marine microbial interactions.

*Salegentibacter* sp. strain BDJ18 was isolated from seawater that had been collected at the deep chlorophyll maximum (55 m; salinity, 36.8 practical salinity units [PSU]; 23°C) in the northeast Atlantic Ocean (36.96294°N, −71.21921°W) onboard the R/V *Neil Armstrong* in May 2017 (cruise AR16). Plankton-associated bacteria were grown by filtering seawater onto a 5-μm-pore-size filter and stamping it onto an F/2 agar plate (1% agar with filtered seawater, tryptone, yeast extract, and F/2 nutrients [3]). Colonies were assayed on chrome azurol S plates (4), and those with halos indicating siderophore production were restreaked and maintained on F/2 plates at 25°C. The *Salegentibacter* sp. strain BDJ18 colonies were yellow and were stored in 30% glycerol at −80°C in June 2017.

For genomic DNA isolation, BDJ18 was revived from a glycerol stock and grown in the aforementioned F/2 medium without agar. DNA was purified with the NucleoSpin DNA RapidLyse kit (Macherey-Nagel, Düren, Germany), quantified with a Qubit fluorometer (Invitrogen, Waltham, MA, USA), and sheared with an ultrasonicator (Covaris, Inc., Woburn, MA, USA). Sanger sequencing of the PCR-amplified 16S rRNA gene was used to identify the isolate as *Salegentibacter* sp. The sequence library was prepared by the Rhode Island Genomics and Sequencing Center (Kingston, RI, USA) using an Apollo next-generation sequencing (NGS) library preparation system with the PrepX DNA library kit (TaKaRa Bio USA, Inc., Mountain View, CA, USA), run on a Bioanalyzer DNA high-sensitivity chip (Agilent Technologies, Inc., Santa Clara, CA, USA), and quantified by quantitative PCR (qPCR) in a LightCycler 480 system (Roche Molecular Systems, Inc., Pleasanton, CA, USA) with an Illumina kit (KAPA Biosystems, Woburn, MA, USA). Samples were sequenced (2 × 300 bp) with the 600-cycle reagent kit on a MiSeq system (Illumina, Inc., San Diego, CA, USA), yielding 2,614,628 paired-end reads. Paired-end reads were uploaded to the open-source U.S. Department of Energy Systems Biology Knowledgebase (Kbase) (http://kbase.us) (5), where Trimmomatic v1.2.14 (6) was used to remove NexteraPE-PE adapters (2 seed mismatches, 30 palindrome clip, and 10 simple clip) and to perform quality filtering (4-bp sliding window with 15 minimum quality, 20 leading and trailing minimum quality, and 20 bp minimum). SPAdes v1.2.4 (7) with default settings was used to assemble contigs, with coverage ranging from 19× to 774×, after removal of contigs with <1,000 bp or with zero coverage. The PATRIC v3.6.8 Similar Genome Finder (8) identified *Salegentibacter* sp. strain T436 (GenBank accession number PRJNA297197) as a good reference genome at a distance of 0.3937, supporting the designation of BDJ18 as *Salegentibacter.* The MAUVE Contig Mover (9) in Geneious Prime v2021.0.1 ordered the BDJ18 contigs against *Salegentibacter* sp. strain T436. In Kbase, *Salegentibacter* sp. strain BDJ18 was assessed with CheckM v1.4.0 (10) and QUAST v0.0.6 (11). Annotations were performed using web-based RASTtk (https://rast.nmpdr.org/; February 2021) with automatic error correction, as *Salegentibacter* sp. (NCBI taxonomy identifier 903072) (12–14). FeGenie v1 (15) identified potential iron-related genes, and antiSMASH v5.0 (16) predicted specialized metabolites.

The *Salegentibacter* sp. strain BDJ18 genome is 3,847,815 bp, with 41 contigs and a GC content of 36.87%. It is 99.4% complete, with an *N*_50_ value of 177,704 bp (10, 11). It has 3,510 coding sequences and 45 RNAs across 264 subsystems. Potential genes include those for resistance to the antibiotic fluoroquinolone and transport of the siderophore enterobactin. Bacterium-phytoplankton interaction genes include those for potential auxin biosynthesis, which may increase phytoplankton growth (17), and those for mitigation of oxidative stress, providing possible protection from phytoplankton-derived reactive oxygen species (18). Only three potential biosynthetic gene clusters (a type III polyketide synthase [PKS] system, arylpolyene, and terpene) were identified, suggesting a limited number of modular biosynthetic systems. This draft genome increases knowledge of how marine bacteria are equipped to interact with other microbes.

### Data availability.

This whole-genome shotgun project has been deposited in DDBJ/ENA/GenBank under the accession number JAFLQX000000000. The version described in this paper is version JAFLQX010000000. The associated raw sequencing reads have been deposited under the SRA accession number SRR13857245 under the BioProject accession number PRJNA706513.
